# Case report: First case of fungemia caused by *Papiliotrema laurentii* in a patient with SARS-CoV-2 infection

**DOI:** 10.1590/0037-8682-0480-2024

**Published:** 2024-02-05

**Authors:** Regielly Caroline Raimundo Cognialli, Gabriella Felber Fratucci, Bruno Hassunuma Carneiro, Karyna Leal Antonio, Morgana Ferreira Voidaleski, Larissa Molina Favarello, Vania Aparecida Vicente, Flávio Queiroz-Telles

**Affiliations:** 1 Universidade Federal do Paraná, Hospital de Clínicas, Curitiba, PR, Brasil.; 2 Universidade Federal do Paraná, Programa de Pós-Graduação em Microbiologia, Parasitologia e Patologia, Curitiba, PR, Brasil.; 3 Universidade Federal de São Paulo, Escola Paulista de Medicina, São Paulo, SP, Brasil.; 4 Universidade Federal do Paraná, Departamento de Patologia Básica, Curitiba, PR, Brasil.; 5 Universidade Federal do Paraná, Hospital de Clínicas, Departamento de Saúde Coletiva, Curitiba, PR, Brasil.

**Keywords:** Papiliotrema, fungemia, SARS-CoV-2

## Abstract

Previously considered saprobe and non-pathogenic, the fungus *Papiliotrema laurentii* (formerly known as *Cryptococcus laurentii)*, is rarely associated with human infection. Nevertheless, there has been an increase in reported infections by non-*neoformans* cryptococci. After a literature search on the Cochrane Library, LILACS, SciELO, MEDLINE, PubMed, and PMC (PubMed Central) databases, we conclude that this is the first case report of fungemia and probable meningitis caused by *Papiliotrema laurentii* in a previously immunocompetent host with associated COVID-19.

## INTRODUCTION

*Papiliotrema laurentii*, formerly known as *Cryptococcus laurentii*, is rarely associated with human infection^1^. It can be found in the environment and has been previously considered a saprobe and non-pathogenic fungus^2^. However, in recent decades, the number of reported cases of infections caused by non-*neoformans* cryptococci, mainly *Papiliotrema laurentii* and *Naganishia albida* (formerly *Cryptococcus albidus*), has increased^1^ ^-^ ^3^. Fungemia caused by non-*neoformans* cryptococci has been more commonly reported in immunocompromised hosts, such as patients with HIV infection, lymphoproliferative disorders, malignancy, sarcoidosis, users of corticosteroid therapy, and after solid-organ transplantation^2^ ^-^ ^5^. Since the emergence of SARS-CoV-2, several reports have raised awareness of the association of COVID-19 with invasive fungal infections, including aspergillosis and candidiasis^6^. 

A literature review was carried out in Cochrane Library, LILACS, SciELO, MEDLINE, PubMed, and PMC (PubMed Central) databases. We report the first case of fungemia caused by *Papiliotrema laurentii* in a previously immunocompetent patient with associated SARS-CoV-2 infection.

## CASE REPORT

A 54-year-old Brazilian female patient, with a history of obesity, type 2 diabetes mellitus, heart failure, hypertension, and hypothyroidism presented to the emergency department with an 8-day history of cough, dyspnea, myalgia, fever, headache, and fatigue. She developed respiratory failure and underwent orotracheal intubation. A chest computed tomography scan (CT) revealed bilateral ground-glass opacities and consolidation in the left lower lobe. She received intravenous dexamethasone (6 mg/day), ceftriaxone, azithromycin, and prophylactic subcutaneous enoxaparin. SARS-CoV-2 was detected in a nasopharyngeal swab sample by reverse-transcription polymerase chain reaction (RT-PCR). The results of admission blood tests are summarized in Table 1.

**TABLE 1:** Laboratory results from admission blood tests.

Blood tests	Results
Leukocyte count (cells/mm³)	11,860
Lymphocytes (cells/mm³)	593
Platelets (cells/mm³)	265,000
D-Dimer level (mg/L)	1.8
Fibrinogen level (mg/dL)	485
Lactate dehydrogenase level (U/L)	778

**FIGURE 1:**
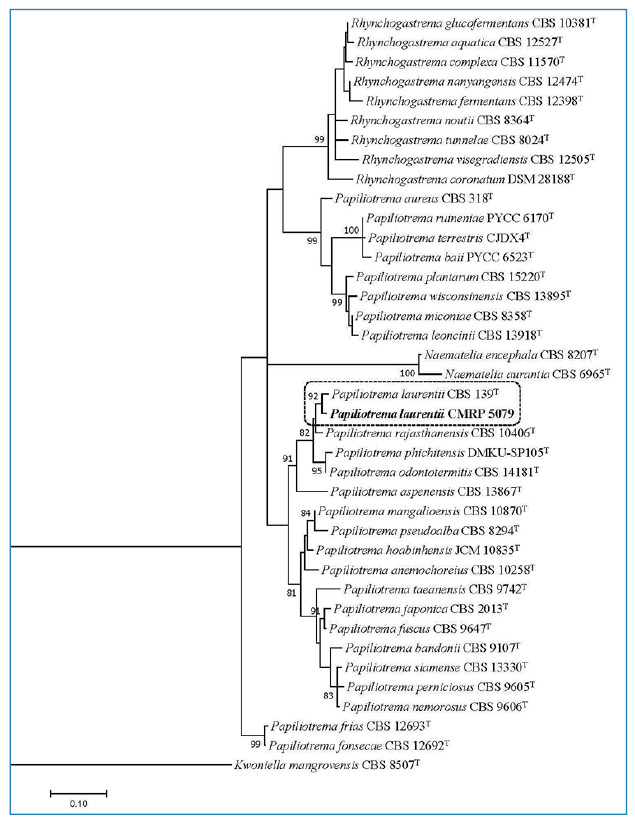
Phylogenetic tree was inferred using the maximum likelihood method based on the Tamura-Nei model with gamma distribution in the MEGA 7.0.26, and bootstrap support was calculated from 1000 replicates. Bootstrap values ≥ 80% were considered statistically significant. The sequencer of ITS region and the D1/D2 region of the LSU rRNA gene showed the isolate CMRP 5079 was close to *Papiliotrema laurentii* CBS 139 type strain. *Kwoniella mangrovensis* CBS 8507T was taken as outgroup. (T) = type strain of the species or of one of its synonyms.

Eleven days after admission, the patient developed severe acute kidney injury and shock; blood cultures were obtained, and antibiotics were changed. Initially, the regimen was changed to piperacillin-tazobactam and then, due to lack of improvement, escalated to meropenem plus vancomycin. After 72 h of incubation, the cultures were positive for yeasts, and micafungin (100 mg/day) was added to the anti-infective therapy. The yeasts were identified afterwards as *Papiliotrema laurentii.* The identification methods are summarized in Table 2. The species was confirmed as *Papiliotrema laurentii* by sequencing the large subunit (LSU) rRNA gene and internal transcribed spacer (ITS) region and clustered with *P. laurentii* CBS139, as shown in the phylogenetic analysis (Figure 1)^7^. The isolate was deposited in the Microbiological Collections of the Paraná Network - Taxonline (CMRP) as *Papiliotrema laurentii* CMRP 5079. Antifungal susceptibility tests were performed using the microbroth dilution method according to the Clinical and Laboratory Standards Institute (CLSI) (Table 2). A total of 15 blood cultures were collected during hospitalization. *Papiliotrema laurentii* was isolated from two independent blood cultures, with a difference of 6 days between each other (on days 11 and 17). At the time of the collection of the first positive blood culture, the patient had leukocytosis (18.980 cells/mm³) and neutrophilia (17.272 cells/mm³). In order to rule out central nervous system involvement, head CT and lumbar puncture were performed. Imaging was unremarkable, and cerebrospinal fluid (CSF) analysis revealed pleocytosis (9 cells/mm³) with lymphocyte predominance (88%), hyperproteinorrachia (377 mg/dL), and glucose and lactate levels of 107 mg/dL and 3.38 mmol/L, respectively. The CSF opening pressure was normal, no microorganisms were seen on direct microscopy, and cultures were negative. Despite negative microbiological results in the initial CSF analysis, induction therapy for severe-disseminated disease was initiated based on the presence of fungemia and lymphocytic meningitis with fluconazole (FLC) (800 mg/day) plus amphotericin B (AmpB) deoxycholate (50 mg/day). Serial blood cultures and CSF samples were drawn for treatment monitoring; follow-up blood cultures were negative, CSF leukocyte count normalized (0 cell/mm³) after 1 week, and CSF protein levels reduced to 77 mg/dL after 2 weeks of antifungal therapy. Induction therapy with FLC plus AmpB was prescribed for 2 weeks, and consolidation therapy with FLC (800 mg/day) was maintained for 2 months afterwards. FLC (300 mg/day) was indicated for maintenance therapy, and the patient was discharged with oxygen support via tracheostomy after a 3-month hospitalization.

**TABLE 2:** Identification of Papiliotrema laurentii CMRP 5079 and antifungal susceptibility tests.

Yeast identification				Results					
Time of incubation for growth in BACTEC Plus Aerobic/FBD culture medium (Becton-Dickinson, Sparks, USA)				72h					
Colonies on blood agar				Smooth and cream colored					
Nigrosin stain				Round-to-oval yeasts with slight capsule					
Temperature of growth				30 °C optimum growth					
				37 °C poor growth					
Urease				Positive					
Niger media				No melanin pigment					
Vitek2 (bioMerieux, Durham, NC)				*Cryptococcus laurentii*					
MALDI-TOF Vitek MS (bioMerieux, Durham, NC)				*Cryptococcus laurentii*					
Cryptococcal antigen lateral flow assay in blood (CrAg LFA; IMMY Inc., Norman, OK)				Negative					
Cryptococcal antigen latex agglutination Pastorex Crypto Plus in blood (Bio-Rad, Marnes-la-Coquette, France)				Negative					
LSU rRNA and ITS region sequencing				*Papiliotrema laurentii*					
	**Antifungal susceptibility tests for *Papiliotrema laurentii* CMRP 5079 isolates**								
	Amphotericin B			Fluconazole			Voriconazole		
	24h	48h	72h	24h	48h	72h	24h	48h	72h
*P. laurentii* CMRP 5079 I (MIC, μg/mL)	0.25	0.25	0.5	4	8	8	0.125	0.25	0.125
*P. laurentii* CMRP 5079 II (MIC, μg/mL)	0.25	0.25	0.5	4	8	8	0.125	0.25	0.125

Legend: MIC: Minimal Inhibitory Concentration; LSU rRNA: Large subunit ribosomal ribonucleic acid; ITS: Internal Transcribed Spacer

## DISCUSSION

Cryptococcal infections are caused by encapsulated yeast-like fungi of the genus *Cryptococcus*; the most common species complexes are *C. neoformans* and *C. gattii*. These microorganisms are typically found on soil contaminated with pigeon excreta and trees. Infections caused by *P. laurentii* are relatively uncommon; they are more frequently described in immunocompromised patients^2^ ^-^ ^5^. Risk factors for infection include impairment of cell-mediated immunity, HIV/AIDS, hematologic malignancies, neutropenia, corticosteroid treatment, chemotherapy, solid-organ transplantation, impaired humoral immunity, non-HIV-related lymphopenia, and direct or indirect exposure to pigeon excreta. Clinical manifestations include skin and ocular lesions, lung infection, peritonitis, and meningitis. Patients can also present with systemic symptoms such as fever and septic shock. Most fungemia cases have been reported in previously immunocompromised patients^5^. 

SARS-CoV-2 infection can be associated with different levels of immunosuppression. The immunosuppression induced by moderate-to-severe COVID-19 is aggravated by exposure to corticosteroids and invasive devices, which may predispose individuals to infection by uncommon pathogens, such as *P. laurentii* ^6^ ^,^ ^8^. Cryptococcemia has been associated with COVID-19 during the use of immunosuppressive agents and corticosteroids^8^ ^,^ ^9^. Other fungal infections have been described in COVID-19 patients, including pulmonary aspergillosis, invasive candidiasis, and mucormycosis^6^ ^,^ ^9^. In 2022, a case of endogenous endophthalmitis caused by *P. laurentii* was described in a post-COVID-19 patient who had received corticosteroid therapy^10^. 

Evidence guiding the treatment of non-*neoformans* cryptococcal infections is limited by the paucity of cases and the lack of clinical studies. For *P. laurentii* bloodstream infection, AmpB has been used in several cases, mostly in association with flucytosine and FLC^2^. Monotherapy with FLC has also been successful in some patients with fungemia^3^. Similarly to fungemia, data available on the management of meningitis are limited to case reports. The reported duration of therapy ranges from 14 days in patients with fungemia to 36-150 days in patients with meningitis and endophtalmitis^10^. Nevertheless, the treatment strategies used in this case were in accordance with clinical practice guidelines for the management of severe-disseminated cryptococcal disease^11^. Although *P. laurentii* CMRP 5079 showed reasonably high FLC minimal inhibitory concentrations (MIC) *in vitro* (4-8 μg/mL), resistance *in vivo* was not observed, as the patient had favorable clinical and microbiological outcomes. It is important to note that there are currently no interpretative breakpoints available for *Cryptococcus/Papiliotrema* spp. regarding FLC resistance^12^.

In conclusion, the present study describes the first case of fungemia and probable meningitis caused by *P. laurentii* in a previously immunocompetent patient with COVID-19. Considering the vulnerability of patients with severe COVID-19 to coinfections, early suspicion and detection of fungal pathogens are necessary to reduce morbidity and mortality.
